# Evaluating Cosmetic Efficacy: A Comparative Study on Methods for Assessing Skin Redness Reduction and Soothing

**DOI:** 10.1111/jocd.71100

**Published:** 2026-08-13

**Authors:** Yue Du, Jingjing Li, Qiwen Sun, Chao Zhang, Feifei Wang

**Affiliations:** ^1^ Botanee Bio‐Technology Group Co. Ltd. Kunming Yunnan China; ^2^ Yunnan Characteristic Plant Extraction Laboratory Co. Ltd. Kunming Yunnan China; ^3^ Shanghai Jiyan Bio‐Pharmaceutical Co. Ltd. Shanghai China

**Keywords:** clinical efficacy test, cosmetics, redness reduction and soothing, vitro test

## Abstract

**Background:**

The growing population affected by skin redness, driven by environmental changes and lifestyle factors, underscores the need for effective cosmetic solutions. While such products can significantly alleviate physical discomfort and improve psychological well‐being, a lack of standardized technical guidance persists for laboratory methods assessing their redness‐reducing and soothing efficacy.

**Objectives:**

This study compared widely used in vitro models—including cell assays, 3D skin models, and the zebrafish model—with clinical efficacy tests to evaluate the applicability and reliability of laboratory detection methods for the redness reduction and soothing efficacy of cosmetics.

**Methods:**

A HUVEC cell model was established to determine the levels of erythema relief‐related factors, including ET‐1, PGI2, and VEGF. A reconstructed 3D skin model was constructed to examine epidermal thickness, dermal‐epidermal junction (DEJ) status, and the area of vascular‐like structures. A zebrafish inflammatory model was applied to detect blood flow velocity. In the human clinical trial, a 28‐day product application test was performed, and transepidermal water loss (TEWL) and cheek redness area were measured on day 14 and day 28, respectively.

**Results:**

The findings indicated that not all products exhibit efficacy in the metrics assessed via cellular and 3D skin models; nevertheless, a comprehensive analysis of the holistic metrics reveals that the outcomes have high rank‐order correlation with those derived from clinical trials. Among all models, the results obtained from the zebrafish model had the highest correlation to clinical efficacy studies.

**Conclusion:**

These findings confirm that the evaluated laboratory methods can be effectively and reliably applied to redness reduction and soothing efficacy of cosmetics. They not only provide efficient and credible tools for efficacy verification during cosmetic research and development, but also offer novel options for alleviating clinically relevant cutaneous symptoms and guiding daily skincare selection.

## 1 Introduction

As the largest organ of the human body, the skin covers the entire body and directly interacts with the external environment. It performs multiple physiological functions such as physical barrier, immune defense, body temperature regulation, and sensory transduction [1]. Skin sensitivity with redness refers to an excessive response of the skin to low‐intensity external stimuli under physiological or pathological conditions, characterized objectively by erythema and often accompanied by subjective discomforts such as burning, stinging, and itching [2, 3]. In recent years, due to factors such as environmental pollution, skincare habits, and individual physical differences, the incidence of skin sensitivity accompanied by redness increased annually. This issue not only impairs skin health but may also trigger psychological problems such as anxiety and low self‐esteem, seriously reducing the quality of life [4].

In 2021, the International Skin Care Association (ISCA) defined skin sensitivity with redness as “a syndrome based on abnormal skin barrier function or neurovascular hyperreactivity, co‐induced by endogenous and exogenous factors, and involving the imbalance of the barrier‐nerve‐vascular‐immune network.” This definition emphasizes its characteristic of multi‐system involvement and highlights the core features of “hyperreactivity” and “multi‐factor interaction.” Current research indicates that the mechanisms underlying sensitivity with redness primarily consist of four aspects [5]:

- Impaired skin barrier: Impairment of the skin barrier leads to incomplete stratum corneum structure, increased epidermal permeability, and unbalanced intercellular lipid content—making the skin more susceptible to stimuli and resulting in redness [6, 7].
- Abnormal cutaneous sensory nervous system: The abnormality is typically associated with the transient receptor potential vanilloid 1 (TRPV1) receptor, which transmits sensations such as pain, burning, pruritus, and other stimuli [8].
- Increased vascular reactivity: Abnormalities of the vascular system serve as the direct driving mechanism of skin erythema. Stimulation via the nervous system and secretion of inflammatory factors induce vascular endothelial cells to synthesize growth factors and vasodilators, which heighten vascular reactivity and consequently trigger skin flushing [9].
- Immune‐inflammatory responses: Abnormal activation of the immune system can exacerbate barrier damage and vasodilation by releasing inflammatory factors, resulting in persistent redness [10, 11].

To alleviate the discomfort caused by sensitive skin, the Regulations on Classification and Catalog of Cosmetics defines “soothing” as a function that “helps improve skin irritation and related conditions” [12]. Thus, scientific evidence supporting the soothing effect claimed by cosmetics is particularly critical. However, current evaluation methods primarily focus on phenotypes such as pain, heat, and itching, lacking direct evaluation of skin redness. This study assessed the redness‐reducing and soothing efficacy of cosmetics through cell model, 3D skin model, and zebrafish model experiments in a more direct and multidimensional way. Finally, the results were analyzed and compared with outcomes of clinical trial in order to confirm the applicability and reliability of the methods.

## 2 Materials and Methods

### 2.1 Cell Culture

Human immortalized umbilical vein endothelial cell (HUVEC‐SV40) was sourced from SiMotai (Shanghai) Biotechnology Co. Ltd. The cells were cultured in primary endothelial cell‐specific medium, maintained at 37°C, 5% CO_2_, and saturated humidity in a CO_2_ incubator. When the cells confluence reached approximately 80%, they were subcultured at a ratio of 1:2. During subculture, the original medium was aspirated, and the cells were washed once or twice with PBS buffer. Then, 0.125% trypsin was added, and the cells were placed in the incubator for digestion for 1 min. An equal volume of medium was added to terminate digestion. The cells were centrifuged at 1000 rpm for 5 min, then the pellet was collected for subculturing.

### 2.2 Establishment of TNF‐α‐Induced HUVEC Model

HUVEC‐SV40 cells in logarithmic growth phase with good viability were seeded into 24‐well plates at a density of 4.0 × 10^4^ cells/well (500 μL per well), then cultured for 24 h at 37°C, 5% CO_2_, and saturated humidity. After aspirating the original medium, four groups were set up: control group, model group, positive drug group, and sample group. Control group and model group added primary endothelial cell‐specific medium. Positive drug group added primary endothelial cell‐specific medium containing positive drugs 40 μM Aspirin (MedChemexpress, HY‐14654R) or 80 μM Dexamethasone (Sigma, US). Sample group added primary endothelial cell‐specific medium containing the sample (at a concentration that maintained ≥ 90% cell viability). After 1 h of pretreatment, inducer TNF‐α (Peprotech 300‐01A) was added to the model group, positive drug group, and sample group to reach a final concentration of 40 ng/mL. The cells and supernatants were collected after further incubation for 24 h.

### 2.3 RT‐PCR of HUVEC Cells

Total RNA was extracted by kit from Invitrogen (ThermoFisher Scientific) and the whole process followed the protocol provided by the manufacturer. The RNA concentrations of different samples were qualified according to the 260/230 and 260/280 ratios. cDNAs were synthesized by reverse transcription using a kit from Takara Biomedical Technology. Quantitative PCR was executed using SYBR qPCR Mix (Toyobo, Osaka, Japan), specific primers, and the synthesized cDNA. The thermal cycling protocol consisted of 45 cycles: denaturation (95°C, 15 s), annealing (62°C, 60 s), and extension (72°C, 60 s); the whole assay was conducted with LightCycler 96 (Roche). The ET‐1 expression was normalized to glyceraldehyde‐3‐phosphate dehydrogenase (GAPDH), and the relative expression levels were computed using the *R* = 2 − [ΔΔCt] method. Primers used were as follows: ET‐1 (forward) 5′‐TCCAAAATCAAGTGGGGCGA‐3′ and (reverse) 5′‐AAATGAGCCCCAGCCTTCTC‐3′; GAPDH (forward) 5′‐ACAACCGAGCACATTGGTGA‐3′ and (reverse) 5′‐GGATGCTCCTGCTCTGATCC‐3′.

### 2.4 The 3D Reconstructed Skin

The 3D reconstructed skin and treatment were conducted in collaboration with LABSKIN Creations. A functional 3D atopic reconstructed skin integrating endothelial cells in the dermal compartment was developed and the samples were applied to the 3D skin model in a topical way; each condition was produced in triplicate.

### 2.5 Histological Analysis of 3D Reconstructed Skin

To evaluate the global cutaneous structure of samples, hematoxylin‐phloxin‐saffron (HPS) staining was performed. Paraffin sections of 5 μm of each condition were cut. After dewaxing and rehydration, the samples were stained with HPS. After rinsing, the sections were dehydrated before the mounting of the slides with a hydrophobic mounting medium.

### 2.6 Immunohistological Analysis of 3D Reconstructed Skin

For immunohistochemistry on paraffin sections, after heat‐mediated antigen retrieval treatment, non‐specific binding was blocked in PBS containing 4% BSA. Sections were then incubated with the filaggrin or CD31 primary antibodies diluted in PBS/BSA 4% overnight at room temperature. After incubation for 1 h with EnVision anti‐mouse/rabbit‐HRP secondary antibody (Dako EnVision^+^, HRP), apply DAB^+^ substrate solution to the sections to reveal the color of the antibody staining. Counterstain slides by immersing them in 25% Harris Hematoxylin counterstaining solution. As a negative control, the primary antibody was replaced by the corresponding IgG class.

### 2.7 Zebrafish Rearing Method and Embryo Collection

The adult zebrafish were reared in system water at a temperature of 28.5°C under a 14‐h light/10‐h dark photoperiod cycle and fed twice daily. One female and two male fish were placed in a breeding tank separated by a divider on the evening prior and embryos were collected in the following morning. The collected embryos were then placed in embryo medium and incubated at 28.5°C in an illuminated incubator for subsequent development.

### 2.8 Zebrafish Blood Flow Velocity Test

A sufficient number of 72 hpf zebrafish larvae with consistent developmental status were pre‐selected. The larvae were randomly allocated into 12‐well plates, with 30 larvae per well. The standard dilution water in the wells was carefully removed, and 1 mL of the corresponding test solution was added to each well (Blank control: standard dilution water; induction group: ${\text{CuSO}}_{4}^{+}$; positive control group: ${\text{CuSO}}_{4}^{+}$ dexamethasone; test substance group: ${\text{CuSO}}_{4}^{+}$ test substance). After 2 h of incubation, the test solution was aspirated, followed by rinsing with standard dilution water three times. The zebrafish were then observed and video‐recorded under a stereomicroscope. Using the blood flow analysis system (Noldus DanioScope) to measure the mid‐abdominal segment of each zebrafish, the blood flow velocity in the main vessels was analyzed to measure.

### 2.9 Consumer Research

All consumers were fully informed of the test procedures and provided voluntary written informed consent prior to participation. Participants aged between 18 and 60 years were enrolled in the study. The inclusion participants who were determined to have positive lactic acid irritation during the initial screening, have no other skin diseases or systemic diseases, or are not undergoing any treatment, they wound not use other facial skin care products during the trial period and should avoid excessive exposure to sunlight.

Participants use the experimental sample continuously for 28 days as per the instructions. Prior to product application, as well as 14 and 28 days post, the transepidermal water loss rate (TEWL) is measured by the skin water loss detector, and the facial images were collected using VISIA CR. Subsequently, the area of red skin on the cheeks was analyzed.

### 2.10 Statistical Analysis

Data are presented as the mean ± SE. *p* < 0.05 was considered significant. Statistical analysis was performed using One‐way ANOVA for statistical analysis of all differences among group means. Visual representations of the data, including graphs, were generated using GraphPad PRISM 7.00 (GraphPad Software, USA). Reproducibility was checked by repeating the experiments three or more times for all the tests.

## 3 Results

### 3.1 Assessment of Redness‐Reducing and Soothing Efficacy of Cosmetics With TNF‐α‐Induced HUVEC Cell Model

**FIGURE 1:**
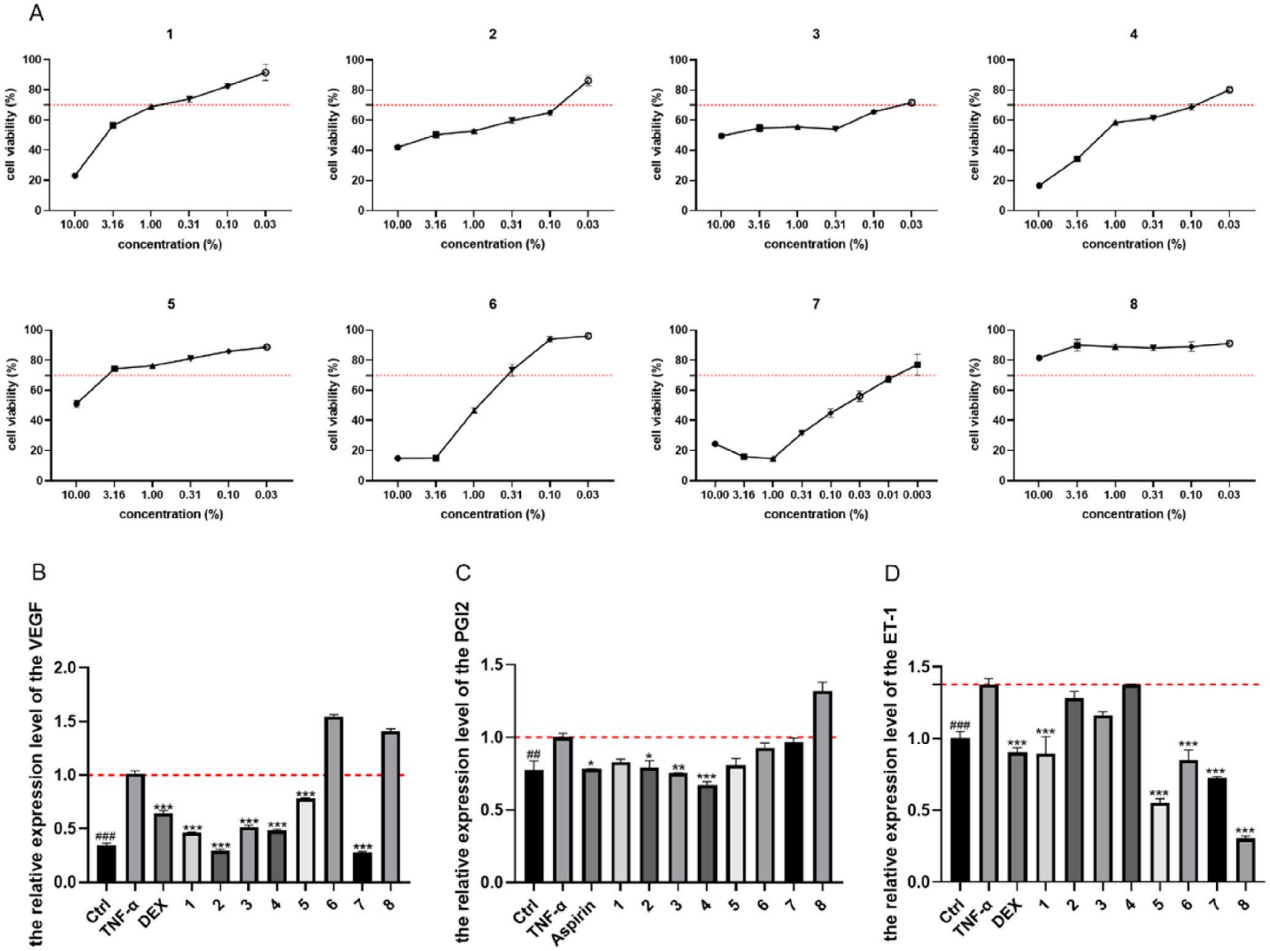
Assessment of redness‐reducing and soothing efficacy of cosmetics using TNF‐α‐induced HUVEC cell model. (A) The results of the HUVEC cytotoxicity assay; (B) the relative expression level of the VEGF factor; (C) the relative expression level of the PGI_2_ factor, and (D) the relative gene expression value of ET‐1. “#” represents the model group compared to the control group; “*” represents the sample group compared to the model group.

Eight products were selected for redness‐reducing and soothing efficacy testing in this study. The results of HUVEC cytotoxicity assay are shown in Figure 1A. The concentrations corresponding to 70% cell viability (CV70) were determined as follows: Winona Barrier Multi‐Effect Special Care Cream CV70 (0.75%); Winona Soothing & Moisturizing Special Care Cream (2nd Generation) (0.08%); Winona Barrier Special Care Cream (0.04%); Winona Soothing Special Care Moisturizing Cream (0.08%); Winona Blue Copper Peptide Repair Essence (4.0%); Winona Clear Complexion Soothing & Calming Cream (0.35%); Winona Baby Moisturizing Cream 2.0 (Moisturizing Version) (0.01%); AOXMED Multi‐Layer Repair Soothing Gel (10.0%). The concentrations used in subsequent efficacy tests were all consistent with the concentrations corresponding to the CV70 (cell viability 70%) values.

VEGF, PGI_2_ and ET‐1 are key factors involved in the mechanism of increased vascular reactivity in sensitive skin. After induction by the inflammatory factor TNF‐α, the expressions of VEGF, PGI_2_ and ET‐1 are significantly upregulated, which promoted angiogenesis and increased vascular permeability, thereby causing vasodilation and persistent skin redness [5, 9]. Among the tested products, Sample 1, 2, 3, 4, 5, 7 significantly downregulated VEGF expression (Figure 1B); Sample 2, 3, 4 significantly reduced PGI_2_ expression (Figure 1C) and Sample 1, 5, 6, 7, 8 significantly reduced ET‐1 expression (Figure 1D).

### 3.2 Assessment of Redness‐Reducing and Soothing Efficacy of Cosmetics With 3D Skin Model

**FIGURE 2:**
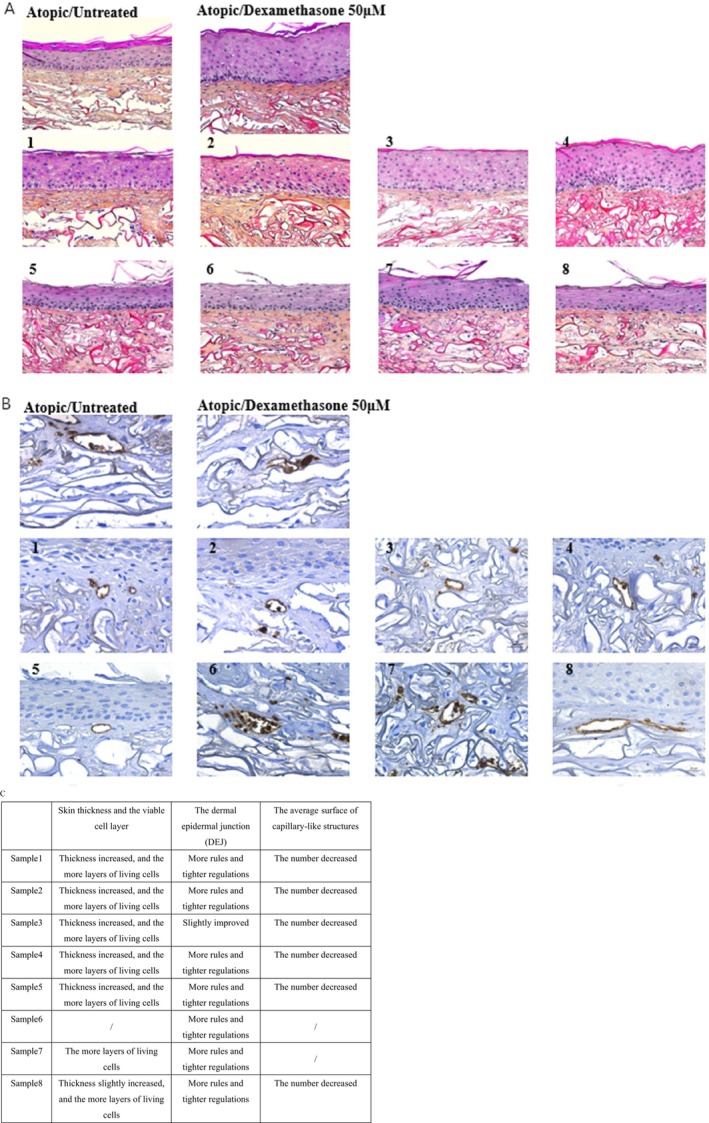
Assessment of redness‐reducing and soothing efficacy of cosmetics using a 3D skin model. (A) The skin thickness and the viable cell layer and the dermal epidermal junction (DEJ). (B) The average surface of capillary‐like structures. (C) Summary table of results for the 8 samples.

Immunohistochemical staining results showed that all test products except sample 6 significantly increased the thickness of 3D atopic skin and the viable cell layers in the epidermis. The dermal‐epidermal junction (DEJ) also appeared more regular after treatment with all products (Figure 2A). In the dermis, the average surface area of capillary‐like structures was markedly reduced for all samples except sample 6 and sample 7, indicating an effective alleviation of skin redness (Figure 2B).

### 3.3 Assessment of Redness‐Reducing and Soothing Efficacy of Cosmetics With Zebrafish Blood Flow Velocity Assay

**FIGURE 3:**
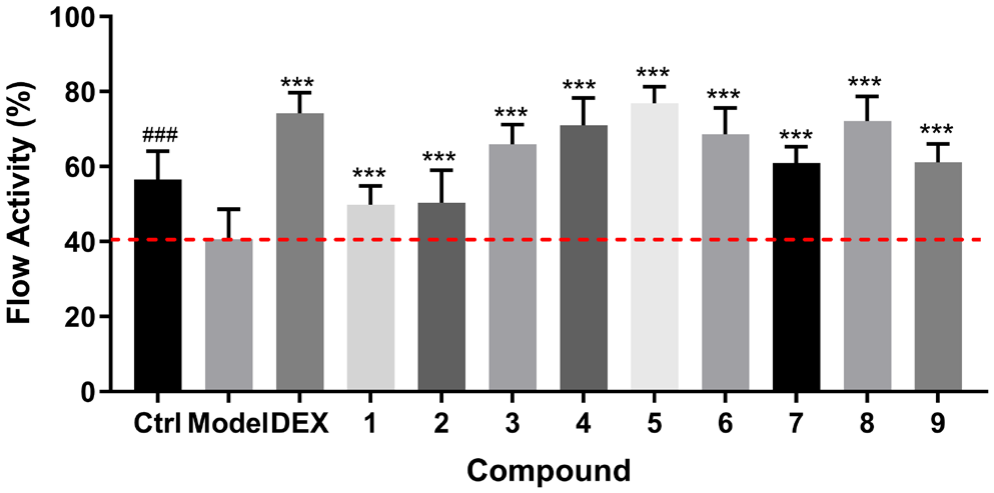
Assessment of redness‐reducing and soothing efficacy of cosmetics using a zebrafish blood flow velocity assay. “#” represents the model group compared to the control group; “*” represents the sample group compared to the model group.

A zebrafish model of vascular dysfunction was established via ${\text{CuSO}}_{4}^{+}$ exposure, which resulted in decreasing blood flow velocity. Hemodynamic impairment was primarily driven by direct endothelial injury, then inflammatory damage occurred. The result clearly indicates the blood flow velocity in the main blood vessels of the model group was significantly lower than that of the control group (*p* < 0.05), and these eight samples were effective in improving vascular function under inflammatory and injury conditions (Figure 3).

### 3.4 Assessment of Redness‐Reducing and Soothing Efficacy of Cosmetics in a Clinical Study

**FIGURE 4:**
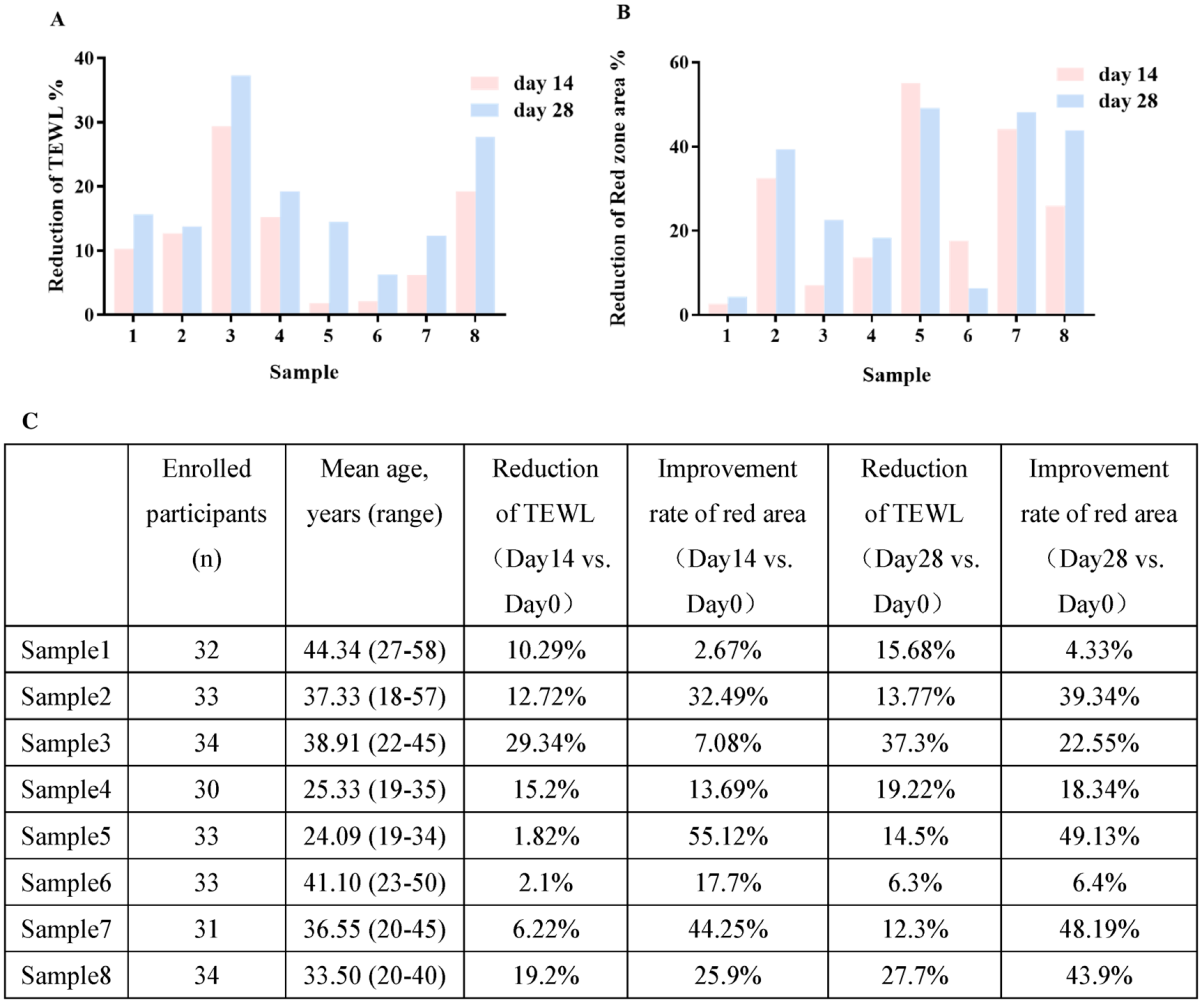
Assessment of redness‐reducing and soothing efficacy of cosmetics in clinical study. (A) The reduction of TEWL after consumers use the sample. (B) The improvement in the area of red zones on consumers' cheeks after using the sample. (C) The number of people included in this clinical efficacy study, their age information, and the specific values of improvement in facial conditions.

The study involved 291 subjects with a mean age of 35.14 years, who completed a 4‐week consumer efficacy research evaluating eight samples. Compared to baseline values, transepidermal water loss demonstrated a decreasing trend after 14 and 28 days of use with all eight samples (Figure 4A). Statistical analysis revealed that the reduction was significant for seven samples at 14 days and for eight samples at 28 days. Analysis of the facial red area quantified by the VISIA Complexion Analysis System showed that, compared to baseline values at Day 0, eight samples demonstrated statistically significant reductions in the percentage of facial red area after 14 days of use. Similarly, eight samples showed significant reductions after 28 days of use (Figure 4B), suggesting a time‐dependent efficacy. Notably, sample 5, sample 7, and sample 8 exhibited particularly pronounced improvements.

### 3.5 Comparative Analysis of Results From Cell Model, 3D Skin Model, Zebrafish Model, and Clinical Trials

**FIGURE 5:**
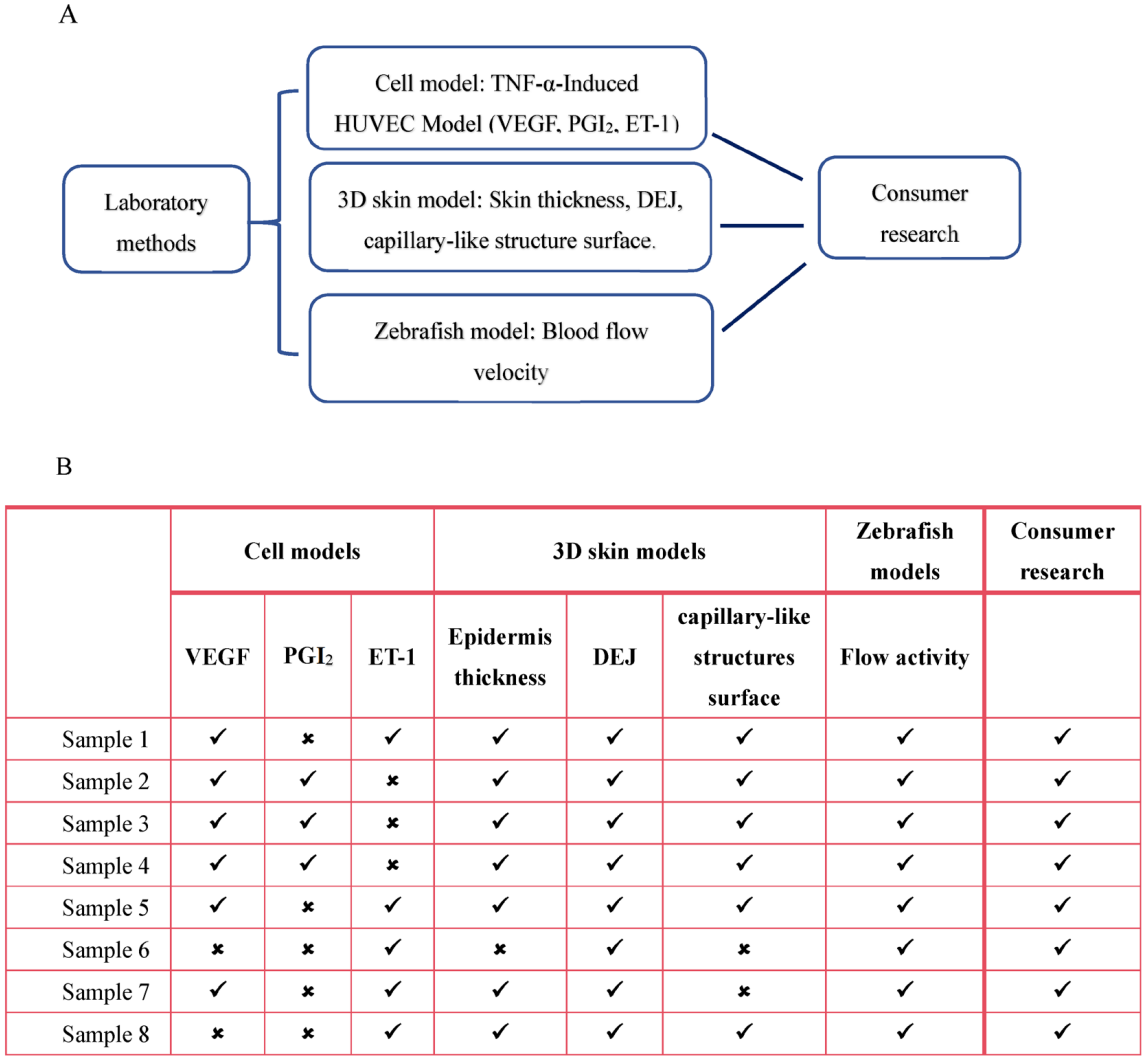
Comparative analysis of results from cell model, 3D skin model, zebrafish model, and clinical trials.

Eight products were tested for their redness‐reducing and soothing efficacy using existing laboratory methods, with the results compared against those from clinical trials (Figure 5A). The findings indicated that not all products exhibit efficacy in the metrics assessed via cellular and 3D skin models; nevertheless, a comprehensive analysis of the holistic metrics reveals that the outcomes have a high rank‐order correlation with those derived from clinical trials. Among all models, the results obtained from the zebrafish model had the highest correlation to clinical efficacy studies (Figure 5B).

## 4 Discussion

Nowadays, human clinical trials play a critical role in cosmetic efficacy evaluation owing to their direct physiological relevance to humans. This methodology combines consumers' subjective sensory feedback with objective instrumental measurements to achieve comprehensive efficacy assessment. Nevertheless, conducting rigorous clinical trials is accompanied by a variety of challenges, including high costs for sophisticated testing equipment, recruitment, screening, and remuneration of qualified participants, as well as difficulties in enrolling an adequate subject cohort and adhering to strict ethical approval protocols. Furthermore, with technological advancements, in vitro assays are increasingly utilized as complementary approaches for efficacy evaluation. This study primarily focused on erythema in sensitive skin, evaluating the redness‐reducing and soothing efficacy of products through laboratory methods. Compared with consumer studies, laboratory assays offered an effective means to verify the applicability of products in humans, with significant savings in labor, materials, and time.

As a 2D univariate model, HUVEC were treated with TNF‐α, a key inflammatory cytokine, to assess the levels of VEGF, PGI_2_, and ET‐1. Elevated VEGF levels trigger microvascular dilation, plasma exudation, and leukocyte infiltration, thereby modeling the erythema and edema characteristic of sensitive skin. The anti‐redness efficacy of the test sample was likely achieved by counteracting pathological vasodilation and blood stasis; this process involved the attenuation of PGI_2_ biosynthesis [13, 14, 15]. The downregulation of inflammation‐induced ET‐1 expression likely contributes to the synergistic anti‐redness and soothing efficacy of the product; this was achieved through multiple mechanisms: by improving microcirculatory dysfunction, inhibiting the release of inflammatory factors, and modulating neurovascular reflexes [16, 17]. It is noteworthy that some experimental results of the cell model remain inconsistent with clinical outcomes. Two potential reasons were identified: First, cells exhibited greater sensitivity to the direct effects of test products—a response that could also reflect higher cellular toxicity. Therefore, the maximum safe concentration (MSC) for cells was selected as the efficacy testing concentration. If the MSC was relatively low, the products might not reach concentrations sufficient to exert their intended effects. Second, erythema in sensitive skin was caused by the complex interplay and combined effects of multiple mechanisms, including those related to the skin barrier, nerves, blood vessels, and immunity. Those processes involved a variety of cells and factors, whereas the cell model (with relatively single variables) could not replicate the comprehensive microenvironment present in 3D skin models or zebrafish models. Therefore, cell‐based systems can serve as preliminary screening indicators for the early stages of project evaluation.

The 3D reconstructed skin model was able to explore the effects of products on the epidermal barrier and dermal blood vessels at the macroscopic level of the skin. Increased epidermal thickness generally reflects the optimized regulation of keratinocyte differentiation and proliferation, and the strengthened tissue structure helps enhance defense against external stimuli and alleviate inflammatory responses [18]. Beyond the epidermis, reinforced DEJ not only counters aging but also likely stabilizes the basement membrane, thereby limiting the permeation of inflammatory mediators and contributing to sustained soothing [19]. Furthermore, the observed increase in capillary‐like structures signified active microcirculatory remodeling; this structural improvement enhanced local perfusion, promoting the delivery of nutrients and the clearance of waste and inflammatory mediators—effects that likely supported the reduction of erythema. The results demonstrated that nearly 90% of the tested products improved skin redness. Notably, for one of the key efficacy indices, the in vitro results correlated with those obtained from human trials. The 3D skin model proved suitable for evaluating final products across all dosage forms and represented the in vitro testing method closest to the consumer research.

The zebrafish was employed as a well‐established model organism with an immune system highly conserved relative to mammals; it offered distinct advantages, including a short reproductive cycle and high transparency for observation, making it particularly suitable for efficacy screening. In this model, CuSO_4_‐induced injury triggers innate immune responses, leading to a phenotype of impaired blood flow. By monitoring changes in blood flow velocity as a readout for the anti‐redness and soothing efficacy of test products, we observed a high rank‐order correlation with the results of consumer research. This high level of consistency was attributed to the zebrafish as an intact organism, which exhibited higher tolerance to compound concentrations. Moreover, blood flow velocity served as a comprehensive physiological endpoint reflecting multi‐level and multi‐target effects, thereby providing a robust and predictive assessment for evaluating therapeutic efficacy.

## 5 Conclusions

In summary, in vitro methods could greatly help the research and development of cosmetic redness relief and soothing raw materials; we propose a tiered testing strategy to optimize the development of anti‐redness and soothing products: The 2D cell model was recommended for use throughout the entire development cycle, from early‐stage screening of active ingredients to intermediate‐phase formula optimization, due to its efficiency, cost‐effectiveness, and suitability for high‐throughput assays. In later stages, however, it is advisable to incorporate more physiologically relevant models, such as the 3D skin model or the zebrafish model, for comprehensive validation of efficacy. This integrated approach balances speed and cost with predictive reliability: the 2D model enables rapid iteration and mechanistic insight, while the advanced models confirm efficacy in a more holistic tissue or organismal context. Such a combination accommodates the evaluation needs of most anti‐redness products, reducing both development costs and time‐to‐market while enhancing the translatability and confidence of preclinical findings.

## 6 Limitations

However, the present study has several limitations. This research centers on laboratory testing methods for the redness‐alleviating and soothing efficacy of cosmetic products. To formalize these methods into standardized technical guidelines, stepwise regression analysis should be implemented. This statistical approach can quantify the relative weight and contribution of each laboratory parameter (such as vascular endothelial growth factor (VEGF) and blood flow velocity) to real clinical outcomes. Nevertheless, the current work predominantly adopts one‐way analysis of variance (ANOVA) for constructing individual models. Restricted sample sizes would distort the models derived from stepwise regression and invalidate corresponding statistical analyses, which represent a major limitation of this paper. Even so, this study addresses a critical practical demand in the development of skin care cosmetics, and can therefore deliver reliable data support and research insights for relevant research in this industry.

## Author Contributions

Y.D., J.L., C.Z. designed the study. Y.D., J.L. and Q.S. performed the experiments and analyzed the data. Y.D., J.L. wrote the manuscript. C.Z., F.W. reviewed and revised the manuscript. All authors reviewed and agreed to the final version of the manuscript.

## Funding

This work was supported by the independent research fund of Yunnan Characteristic Plant Extraction Laboratory (2025YKZY001, 2025YKZY009).

## Ethics Statement

This study was conducted in accordance with the ethical standards of the institutional and national research committee and with the 1964 Helsinki declaration and its later amendments or comparable ethical standards.

## Consent

All participants provided written informed consent prior to inclusion in the study.

## Conflicts of Interest

The authors declare no conflicts of interest.

## Data Availability

The data that support the findings of this study are available from the corresponding author upon reasonable request.
